# Proximate Composition, Bioactive Compounds, and Antioxidant Potential of Wild Halophytes Grown in Coastal Salt Marsh Habitats

**DOI:** 10.3390/molecules27010028

**Published:** 2021-12-22

**Authors:** Yasser A. El-Amier, Walid Soufan, Khalid F. Almutairi, Nouf S. Zaghloul, Ahmed M. Abd-ElGawad

**Affiliations:** 1 Department of Botany, Faculty of Science, Mansoura University, Mansoura 35516, Egypt; 2 Plant Production Department, College of Food & Agriculture Sciences, King Saud University, P.O. Box 2460, Riyadh 11451, Saudi Arabia; wsoufan@ksu.edu.sa (W.S.); almutairik@ksu.edu.sa (K.F.A.); 3 Bristol Centre for Functional Nanomaterials, HH Wills Physics Laboratory, Tyndall Avenue, Bristol BS8 1FD, UK; nouf.zaghloul@bristol.ac.uk

**Keywords:** forage, natural resources, proximate composition, saline habitat, secondary metabolites

## Abstract

Halophytes have been characterized as a potential resource for fiber, food, fodder, and bioactive compounds. Proximate composition, bioactive compounds, and antioxidant activity of five wild dominant halophytes (*Arthrocnemum* *macrostachyum*, *Halocnemum* *strobilaceum*, *Limoniastrum* *monopetalum*, *Limoniastrum* *pruinosum*, and *Tamarix nilotica*) naturally growing along the Nile Delta coast were assessed. The soil supporting these halophytes was sandy to sand-silty, alkaline, with low organic carbon, and relatively high CaCO_3_. *H. strobilaceum* attained the highest moisture content, ash, crude fiber, lipids, and total soluble sugars. *L. monopetalum* showed the highest content of crude protein (18.00%), while *T. nilotica* had the highest content of total carbohydrates. The studied halophytes can be ranked according to their nutritive value as follows: *H.* *strobilaceum* > *L.* *monopetalum* > *A.* *macrostachyum* > *L.* *pruinosum* > *T. nilotica*. *A. macrostachyum* attained the highest amount of Na^+^, K^+^, Ca^2+^, and Mg^2+^. *A. macrostachyum* showed a high content of phenolic compounds, while *H.* *strobilaceum* was rich in tannins and saponin contents. The MeOH extract of *A. macrostachyum* and *H. strobilaceum* exhibited substantial antioxidant activity. The present results showed that the studied halophytes could be considered as candidates for forage production or used as green eco-friendly natural resources for bioactive compounds.

## 1. Introduction

Due to the progressive increase in the global population, the pressure on food, fodder, drugs, and raw materials increased in most countries worldwide, and an estimated 50% increase in yields of the major cultivated lands will be required [1]. At the same time, the agricultural soils are shrinking by about 1–2% in arid and semi-arid areas as a result of soil salinity every year [2]. In these arid countries, reduced precipitation and higher temperatures lead to higher salinity [3] and become the most important factor limiting the growth of conventional crops [4]. Therefore, researcher and scientists search for non-conventional plants that cope or tolerate high saline soils. Saline and salt-affected lands are widely distributed globally and makeup about 10% of the Earth’s terrestrial surface [5]. It is recognized as a vital ecosystem that supports a wide group of unique plants especially those adapted to saline conditions. Wetland conditions play a vital role in the global nutrient and element cycle. Thus, they provide key hydrological benefits, such as flood attenuation, shoreline stabilization, erosion control, and water purification [6].

Halophytes are interesting plants as they grow and develop in harsh habitats with high salt contents, where they developed various morphological, anatomical, and physiological mechanisms to cope with salty conditions [7,8,9]. They are distributed from coastal areas on seas to deserts, which occupy about 8% of the worldwide land area [5]. Halophytes are scattered in the world’s continents except for Antarctica. About 1% of the total flora (1500 species) of the world grows and develops in saline conditions and is referred to as salt-tolerant plants or halophytes [10]. Halophytes (hydro- and xero-halophytes) are extremely salt-resistant plants that usually survive and complete their life cycles in environments containing high salt concentrations [11]. Despite the high salt content in the tissues of halophytes, they can be grown and harvested as raw materials in food, animal fodder, and medicines. Additionally, they are characterized by their content of bioactive compounds, that are potentially useful for therapeutic uses [12] and the food industry as additives [13]. Many research papers have been published dealing with various aspects of the physiology of halophytes [14,15,16], biochemistry, molecular biology [17], ecology, and evolution [18]. All of these papers provided insightful suggestions on the mechanisms underlying the vegetative growth and utilization of halophytes.

Egypt comprises six phytogeographical regions [19], namely: Mediterranean coastal region, eastern desert, western desert, the Sinai Peninsula, Red Sea coastal region, and the River Nile region. The salt-affected lands are particular ecosystems present in almost all of these six phytogeographical regions. Among these regions, the Mediterranean coastal region is a recognized biodiversity hotspot. In Egypt, the saline-affected soils were distributed as coastal salt marshes, inland lakes, oases, and depressions [19]. In the Nile Delta, halophytic flora in the coastal strip plays an important role in protecting ecosystems and maintaining ecological stability due to their adaptation abilities. In these coastal areas, the vegetation of the salt marsh habitat is organized consisting of communities dominated or co-dominated by halophytes, e.g., *Arthrocnemum macrostachyum* (Moric.) K.Koch., *Halocnemum strobilaceum* (pall.) M. Bieb., *Inula crithmoides* (L.) Dumort., *Juncus acutus* L., *J. rigidus* Desf., *Limoniastrum monopetalum* (L.) Boiss., *Suaeda* spp., *Tamarix* spp., *Zygophyllum album* L., and *Nitraria retusa* (Forssk.) Asch. etc. [19,20]. The abundance of these halophytes is varied according to their tolerance to salts and their location or distance from the Mediterranean Sea. The main objective of this study was to evaluate the proximate composition and secondary bioactive compounds of some dominant halophytes naturally growing in the northern sector of the Nile Delta, with relation to soil variables.

## 2. Materials and Methods

### 2.1. Study Area and Plant Samples Collection

Egypt is characterized by its unique position between Africa and Asia. It has long coasts of both the Mediterranean Sea in the north (about 970 km) and the Red Sea in the east (about 1100 km). The Mediterranean coastal land of Egypt has a narrow coastal belt that extends between Sallum eastward to Rafah, with an average width ranging of 20–25 km in a north–south direction [19]. The vegetation of the Mediterranean coastal region of Egypt is considered as one of its major natural resources. Its proper utilization plays a vital role in this region, which is known to have enjoyed prosperity during the Graeco-Roman times. However, the coastal zones of Egypt suffer from several serious problems, including unplanned development, land subsidence, excessive erosion rates, waterlogging, saline water intrusion, soil salinization, and ecosystem degradation [21,22]. The coastal zone is more sensitive due to the effect of the sea as well as climate change. This area has annual precipitation up to 200 mm and average temperature ranges from 15.2–25.9 °C [23].

**Figure 1:**
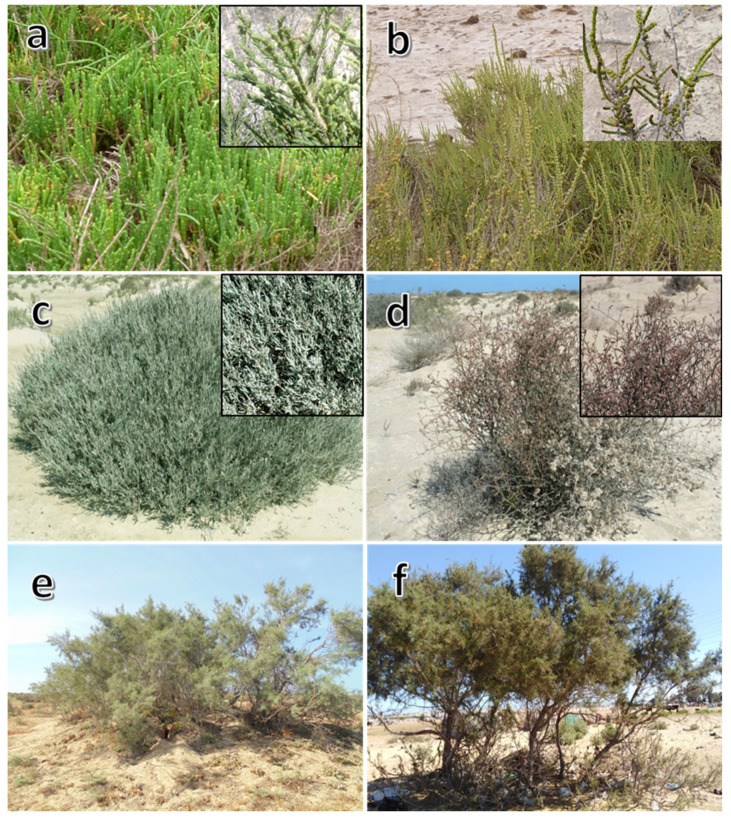
Overview of different studied halophytes. (**a**) Arthrochemum macrostachyum, (**b**) Halocnemum strobilaceum, (**c**) Limoniastrum monopetalum, (**d**) Limonium pruinosum, (**e**,**f**) Tamarix nilotica.

Five halophytes were selected according to their dominance in the study area are (1) two species of Chenopodiaceae (*A. macrostachyum* and *H. strobilaceum*), (2) two species of Plumbaginaceae (*L. monopetalum* and *L. pruinosum*), and (3) one species of Tamaricaceae (*T. nilotica*) (Figure 1).

**Figure 2:**
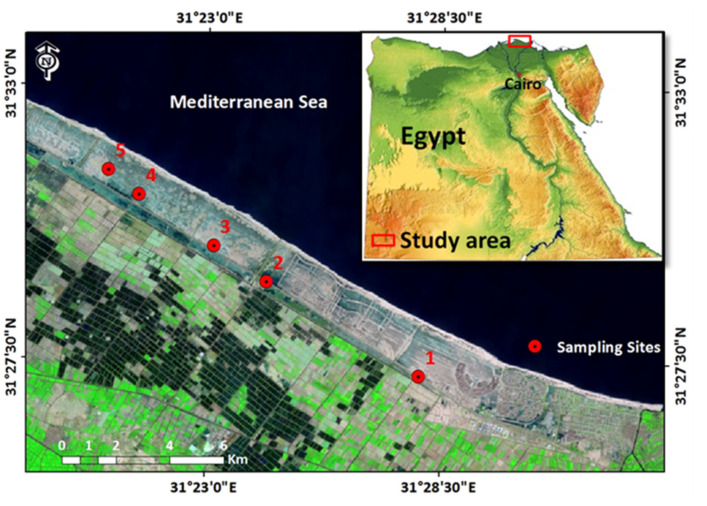
Map showing the different habitats and collection locations of the five halophytes.

The aerial/aboveground parts (stems, leaves, flowers, and fruits) of five wild halophytes were collected in March 2019 (flowering period) from three naturally growing populations distributed along the northern coast of the Mediterranean Sea, Egypt (Figure 2).

From each population, about 10 kg of fresh plant materials were collected from different individuals. The samples were cleaned by hand, washed three times by distilled water to remove dust and other residues, dried at room temperature (25 ± 3 °C) in a shaded place for several days till complete dryness, and ground into powder using a grinder (IKA^®^ MF 10 Basic Microfine Grinder Drive, Breisgau, Germany) at a dimension of 0.5 mm. Finally, the dried samples were stored in paper bags and kept in dark conditions at room temperature until further analyses. Life span, life from, chorotype, and habitats of the studied halophytes species are shown in Table 1.

**Figure 3:**
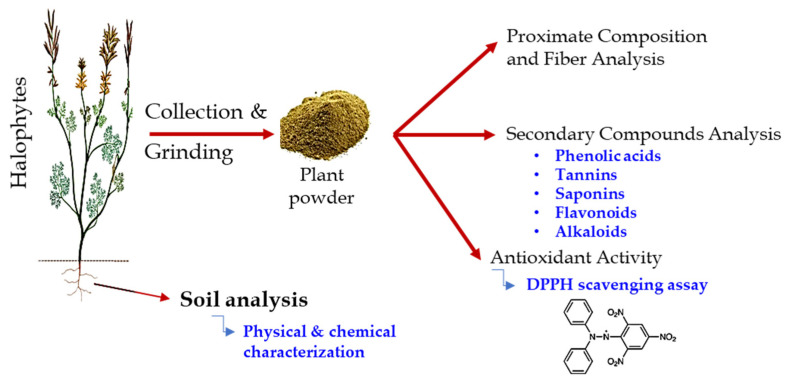
A schematic diagram of the methodological approach.

The plant specimens were collected and authenticated according to Tackholm [24], Boulos [25], and Boulos [26]. Additionally, herbarium sheets (Mans.030113016, Mans.030819013, Mans.161213008, Mans.161216009, and Mans.202014012) were prepared and deposited in the Herbarium of Botany Department, Faculty of Science, Mansoura University, Egypt. Additionally, the healthy aerial parts were collected from three populations of each species. A schematic diagram of the methodological approach is shown in Figure 3.

### 2.2. Soil Analysis

Within each site, soil samples were collected under the three populations at 0–20 cm depth for each halophyte. The soil samples were dried, sieved, and stored until further analysis. Soil particle size (texture), water holding capacity (WHC), soil porosity, organic carbon, and sulphate were determined according to Piper [27]. Chlorides and calcium carbonate content was determined according to Jackson [28]. Soil pH and electrical conductivity (EC) were measured in water suspension (1:2.5), as described by Jackson [28]. Carbonates and bicarbonates were determined by titration using 0.1 N HCl [29]. The extractable cations Na^+^ and K^+^ were determined by flame photometry (PHF 80B Biologie Spectrophotometer, Waltham, MA, USA), while Ca^2+^ and Mg^2+^ were estimated according to Allen et al. [30] using an atomic absorption spectrometer (A Perkin-Elmer, Model 2380, Wellesley, MA, USA). The details were described in our previous work [31].

### 2.3. Proximate Composition Analysis

The moisture content, dry matter, crude fiber, ether extract (lipid), and ash content of selected halophytes were analyzed according to AOAC [32]. The total nitrogen was determined by the Kjeldahl method [33], and protein contents of the plant species were determined by multiplying N contents by the factor 6.25 [32]. Glucose was determined based on the method of Feteris [34]. The lignin content of the plant sample was assessed according to Yuan et al. [35], while the estimation of holocellulose (cellulose + hemicelluloses) content was determined by degrading the lignin polymer according to Allen et al. [36]. About 0.1 g of air-dried sample was submerged overnight in 10 mL of 80% (*v*/*v*) ethanol at 25 °C with periodic shaking. The ethanolic mixture was filtered and the ethanolic filtrate was made up to volume and kept in the refrigerator for analysis of different sugar fractions. Glucose was determined based on the method of Feteris [34]. Sucrose was determined according to van Handel [37]. Total soluble sugars were estimated by the method of Southgate [38]. The total carbohydrate content of plant sample was calculated by “difference”, in this, the sum of the percentages of all the other proximate components was subtracted from 100 [32].

For elements analysis, about 0.1 g of each prepared plant powder was digested with concentrated HNO_3_ with gentle heating till the solution turned clear, and the samples were made up to known volume using dist water. Sodium and potassium were determined using Flame Photometer (Model PHF 80 B Biologie Spectrophotometer, Waltham, MA, USA), while calcium and magnesium were estimated using an atomic absorption spectrometer (A Perkin-Elmer, Model 2380, Wellesley, MA, USA).

### 2.4. Quantitative Estimation of Some Secondary Compounds

The total phenolics content was quantitatively estimated using the Folin–Ciocalteu colorimetric method according to Chlopicka et al. [39]. In brief, 0.1 g of the prepared plant powder was extracted by grinding in CH_3_OH, and centrifugated for 20 min at 10,000 rpm. The supernatant was kept while the residue was extracted again three times, and the supernatants were collected and raised to a known volume. A reaction mixture of 0.5 mL of the extract, 0.5 Folin–Ciocalteu reagent, 2 mL sodium carbonate (20% *w*/*v* in water), and 2.5 mL distilled H_2_O was prepared and vigorously shaken, and kept in a dark condition for 40 min. Then, the absorbance was measured by a spectrophotometer (Spectronic 21D model, Milton Roy, CA, USA) at 725 nm. The phenolics content was assessed upon a standard curve of gallic acid and expressed as mg gallic acid equivalent g^−^^1^ DW.

Tannins were determined spectrophotometrically according to the method of Sadasivam and Manickam [40]. About 1 g of the plant powder was extracted in methanol (80% *v*/*v*) by shaking for 24 h. The mixture was centrifugated and the supernatant was collected, while the residue was re-extracted again three times. The supernatants were collected, pooled, and raised to a known volume. A reaction mixture of 1 mL of the extract and 5 mL of vanillin hydrochloride reagent was incubated at room temperature for 20 min, then the absorbance was measured at 500 nm by a spectrophotometer. Tannins content was assessed regarding a standard curve of tannic acid and expressed as mg g^−^^1^ DW.

Saponin content was estimated according to Obadoni and Ochuko [41]. In brief, a known weight of the plant powder (about 20 g) was mixed with 100 mL aqueous ethanol and heated at 55 °C for 4 h over a water bath. The mixture was filtrated and the filtrate was collected, while the residue was re-extracted three times as mentioned before. The tannins were separated by adding diethyl ether and shaken vigorously in a separating funnel. The aqueous layer was dried over a water bath, where the residue was weighted as total saponins and expressed as mg g^−1^ DW.

The alkaloid was extracted with 10% acetic acid in ethanol and determined according to the method of Harborne [42]. About 1 g of the plant powder was mixed with 40 mL of acetic acid and shaken for 4 h. The mixture was filtrated and the filtrate was collected, while the residue was re-extracted three times. The alkaloids were precipitated using an ammonia solution, dried, and expressed as mg g^−1^ DW.

The total flavonoid content was determined using the aluminum colorimetric method according to Stankovic [43]. The extraction was performed as previously described in the determination of phenolics. About 1 mL of the extract was mixed with 0.3 mL of sodium nitrate and AlCl_3_, and the mixture was kept for 6 min. Then, 2 mL of sodium hydroxide was added, the volume was adjusted to 10 mL by distilled water, and the mixture was kept for 15 min. The absorbance was measured via a spectrophotometer at 510 nm. The total flavonoid was calculated based on a standard curve of rutin and expressed as mg rutin equivalent g^−^^1^ DW.

### 2.5. Antioxidant Activity

According to Miguel [44], the methanolic extracts from the five halophytes aerial parts were tested for antioxidant activity by scavenging 2,2-diphenyl-1-picrylhydrazyl (DPPH•) (Sigma-Aldrich, Darmstadt, Germany). Methanol was employed to prepare various concentrations of methanolic extract (5, 10, 20, 30, 40, and 50 mg mL^−1^). This concentration range was estimated using either a higher or lower concentration in a preliminary test. A reaction mixture comprising equal volumes of newly generated 0.3 mM DPPH• and each concentration of the methanolic extract was prepared, forcefully mixed, and maintained in the dark for 30 min at 25 °C. Additionally, a parallel positive control was performed and treated similarly to the treatments, using ascorbic acid as the standard antioxidant at doses of 1.0, 2.5, 5, 10, 15, and 20 mg mL^−1^. A spectrophotometer (Milton Roy Spectronic 21D UV-Visible Spectrophotometer, Ivyland, PA, USA) was used to measure the absorbance after incubation at 517 nm. The quantity of methanolic extract necessary to decrease DPPH’s absorbance by 50% (SC_50_) was visually estimated.

### 2.6. Statistical Analysis

All proximate composition, secondary compounds, antioxidant activity, and soil analyses were performed in triplicate and the average was calculated. The data were subjected to one way ANOVA followed by Duncan’s post hoc test using CoStat software program (CoHort Software, Monterey, CA, USA).

## 3. Results and Discussion

As the global population increased steadily, there was pressure on food, fodder, drugs, and raw material resources worldwide. In this context, researchers’ and scientists’ attention was directed toward non-conventional resources. Halophytes are interesting plants, growing in harsh habitats with high salt content. Several species of halophytes have been characterized as promising natural resources for fiber, food, fodder, and bioactive compounds.

### 3.1. Properties of Soil Supporting Studied Halophytes

Soil analysis is a set of various chemical processes that determine not only the amount of nutrients in the soil available for plant growth, but also the chemical, physical, and biological soil properties important for soil health [45]. The soil analysis of the represented stands of the five plant samples did not show significant variation (*p <* 0.05) (Table S1). The results elucidated that the soil supporting the growth of the studied plants is sandy to sand-silty in texture with a low amount of clay, with porosity ranged from 38.85 to 43.68%, and a water holding capacity of 37.08–39.81%. Moreover, the soil of all plant samples is generally moist and alkaline (pH = 9.18–9.45).

Organic carbon contents are generally low (0.60–0.75%), and calcium carbonate contents are relatively high, where the greatest (9.09%) and lowest (3.14%) values were found in the soil of *L. monopetalum* and *H. strobilaceum,* respectively. Electrical conductivity, chlorides, sulfates, and bicarbonates were high in the soil of *A. macrostachyum,* while they were low in the soil of *L. monopetalum.* Macro-elements (Na^+^, K^+^, Ca^2+^, and Mg^2+^) content are generally high, particularly in the soil for *A. macrostachyum* (up to 221.85, 34.54, 60.69, and 21.71 mg/100 g DW).

### 3.2. Proximate Composition of the Studied Halophytes

Wild plants are important resources for food, fodder, and livelihoods in smallholder communities and subsistence farming in developing countries [46]. Plant species compositions provide the ecosystem with valuable services and play a crucial role in assessing their nutritional significance for both humans and animals [47]. According to our field observation, the studied five halophytes grow quickly in coastal deserts and do not require fresh water to flourish; instead, they are watered with saline. These plants have a big biomass during most seasons. Therefore, these plants can be considered as natural resources that could be integrated for the production of food, fodder, or pharmaceutical compounds. The proximate analysis of the nutritive contents of the five dominant halophytes is depicted in Table 2. All studied parameters showed significant variation (*p* < 0.001) among the five studied halophytes, except for hemicellulose (*p* = 4.25) and magnesium (*p =* 3.63) concentration.

In the present study, the dry matter content ranged from 79.75% in *H. strobilaceum* to 92.87% in *L. pruinosum*. The dry matter content of plants reveals the actual quantities of various nutrients available for animal consumption [48]. On the other hand, the highest moisture content was determined in *H. strobilaceum* (20.25%) and the lowest (7.13%) was determined in *L. pruinosum*, while *A. macrostachyum*, *T. nilotica*, and *L. monopetalum* attained a moisture content of 16.68%, 11.24%, and 9.11%, respectively. Moisture content is the most vital and usually measured in food processing, storage, as well as it is considered a crucial factor from an economic and food quality point of view. In addition, moisture of food is considered a good source of water, where it represents 20–30% of total water consumption [49]. The ash content ranged between 5.94% in *H. strobilaceum* and 9.58% in *A. macrostachyum*. The ash content of the plant is important as it helps determine the amount and type of minerals in the food [50]. In this context, Al-Rowaily et al. [51] and El-Amier and Al-hadithy [52] documented values on other wild species which are within the range of our study, but lower values were documented by Zahran and El-Amier [53]. The difference in composition could be attributed to the differences in origin, plant species, age, ecological, and climatic factors, or it can be correlated to the physiological status of the plant itself and nutrients available in the soil [54,55,56,57,58].

Fiber fractionation analysis showed highly significant variation (*p < 0.05*) among studied plant species, except for hemicellulose. The crude fiber content of the different species samples varied from 7.92% (*L. monopetalum*) to 22.78% (*H. strobilaceum*). The highest contents of holocellulose (68.97), cellulose (46.61%), and hemicellulose (22.36) were determined in *T. nilotica*, while the lowest contents of holocellulose (51.89%) and cellulose (31.54%) were investigated in *L. monopetalum* (Table 2). Fiber is an important part of the diet for optimal health and the fibrous compounds that reduce the amount of plant material that herbivores can digest [59]. Raw fiber consists largely of cellulose together with a little lignin that enhances digestibility in living organisms [60]. The combination of cellulose and hemicellulose is called holocellulose that usually >65% of the plant’s DW [61]. In the present study, the amount of holocellulose, cellulose, hemicellulose, and lignin were high in *T. nilotica*, while the lowest values were determined in *L. monopetalum, H. strobilaceum,* and *A. macrostachyum*, respectively (Table 2). Ishida et al. [62] reported that foods which are rich in fiber are good for diabetics, reducing blood cholesterol, obesity, and diabetes. A large amount of cellulose (>40%) in plants is suitable for the pulp and paper industry [63]. Data obtained from this study are in harmony with other reported wild species, such as *L. pyrotechnica*, sunflower, date palm leaves, and rice straw [64,65].

Lipid in food is a major source of energy and essential fatty acids [66], and they play a role in the protection of the internal tissues and contribute to important cell processes [67]. The lipid content of the studied halophytes varied from 1.17% in *L. pruinosum* to 5.88% in *H. strobilaceum*. In this context, *T. nilotica*, *A. macrostachyum*, and *H. strobilaceum* showed lipid content of 2.15, 1.45, and 1.41%, respectively (Table 2).

The protein content is considered a building material for bones, muscles, skin, and other tissues in the body. According to Satter et al. [67], 20% of the human body is made up of protein, and plant foods provide more than 12% of its caloric value in protein. In the present study, the protein content of the studied halophytes varied from 5.97% (*T. nilotica*) to 18% (*L. monopetalum*), while *L. pruinosum*, *H. strobilaceum*, and *A. macrostachyum*, attained protein content of 12.81, 12.36, and 6.88%, respectively. The protein content in *H. strobilaceum, L. monopetalum,* and *L. pruinosum* was higher than 10%. This result is in agreement with other reports [51], but it is higher than those reported by Zahran and El-Amier [53] and El-Amier and Al-hadithy [52].

Carbohydrates are the most abundant compounds in living plants. They serve as the main source of energy production in the body and their deficiency causes the depletion of body tissue [68]. The total carbohydrate content was comparable, whereas *T. nilotica* contained relatively higher amounts of total carbohydrates (420.34 mg g^−1^ DW). *H. strobilaceum* attained the highest contents of total soluble sugars (149.51 mg g^−1^ DW) and sucrose (8.31 mg g^−1^ DW), while *L. monopetalum* showed the highest content of glucose (2.65 mg g^−1^ DW) compared to other species (Table 2). The Carbohydrate content of the studied plants is within the range of other reported species [51], but higher than values documented by Zahran and El-Amier [53] and El-Amier and Al-hadithy [52]. Singha and Hassan [58] reported that the type of carbohydrates is more important in the diet than eating large or low amounts. In addition, total carbohydrates in wild plants depend on the type of plant species and its maturity, fibers, and moisture contents, as well as geographical distribution.

To summarize, the studied halophytes can be ranked according to their nutritive value as following: *Halocnemum strobilaceum* > *Limoniastrum monopetalum* > *Arthrocnemum macrostachyum* > *Limoniastrum pruinosum* > *Tamarix nilotica*.

Animals, plants, and microorganisms must have an appropriate chemical balance based on the levels of different minerals in the body for optimal growth and reproduction [69]. The concentrations of the estimated minerals in five studied halophytes are listed in Table 2. The sequence of minerals regarding plant species is *A. macrostachyum* > *H. strobilaceum* > *L. pruinosum* ≈ *T. nilotica* > *L. monopetalum*. Sodium is the primary cation in extracellular fluids in mammals. Our study showed that sodium content was relatively high in all plant species, except *A. macrostachyum* (27.18 mg g^−1^ DW) and *H. strobilaceum* (20.50 mg g^−1^ DW). Underwood and Suttle [70] stated that Mg^2+^ concentration of 0.04% in diets should support maintenance requirements when dietary calcium and phosphorus concentrations are relatively low. The Mg^2+^ content in our study is higher than those reported in white clover [71] and natural grasslands by Vejnovic et al. [72]. According to soil and climatic factors (light, temperature, water, and humidity), plants might be poor or rich sources of minerals [73].

### 3.3. Secondary Metabolites

Plants growing in harsh conditions are usually rich in bioactive compounds. Abiotic stress, including salinity stress, triggers the synthesis of various secondary metabolites, such as phenolics, flavonoids, alkaloids, saponins, and many other compounds [9,74]. These compounds play a vital role in the protection of plants cells [74,75]. The present results show that the content of phenolics, alkaloids, flavonoids, saponins, and tannins were significantly varied among the five studied species (Table 3).

The highest content of phenolics (41.83 mg g^−1^ DW) and flavonoids (8.23 mg g^−1^ DW) was determined in *A. macrostachyum*. The high content of phenolic compounds could be ascribed to the high salinity content in the habitat of this plant (Table S1). The Portuguese ecospecies of *A. macrostachyum* showed phenolic contents of 72 mg g^−1^ DW [76]. The Portuguese ecospecies of *A. macrostachyum* showed phenolic contents of 72 mg g^−1^ DW [76]. However, the Algerian ecospecies of this halophyte has been reported to have a low content of phenolics [77]. Salinity stress has been known to trigger the plant metabolism to produce phenolics as a defense mechanism [78]. This observation has been reported by Al-Rowaily et al. [51], where *Cyperus conglomeratus* that grow in saline sandy habitats attained the highest content of phenolics and flavonoids, while the grasses *Elymus farctus*, *Lasiurus scindicus*, and *Panicum turgidum*, that grow in sandy habitat attained the lowest content.

It is well known that there is a wide variation in the chemical composition among different plant species and among different organs of the same plant. Additionally, the chemical composition varied within plants from different geographic locations, ages, climate, and soil conditions. It is worth mentioned here that *A. macrostachyum* has been reported to have several phenolic acids, such as chlorogenic acid, gallic acid, protocatechuic acid, p-coumaric acid, rosmarinic acid, and caffeic acid [60]. *A. macrostachyum* was traditionally used as antibiotic, alexipharmic, and hypoglycemic agents [7,77,79,80].

On the other hand, *H. strobilaceum* attained 22.29 mg g^−1^ DW of saponins 22.38 mg g^−1^ DW of tannins, while *L. pruinosum* showed the highest content of alkaloids (7.36 mg g^−1^ DW) compared to other studied species (Table 3). Several studies showed that *H. strobilaceum* contains phenolic acids, quercetin derivatives, icaritin, and several glycosides [81]. Therefore, *H. strobilaceum* has been characterized to possess antioxidant, anticancer, and chemopreventive activities.

*Tamarix nilotica* has been used as an antiseptic agent, aphrodisiac aperient, sudorific, ulcer, expectorant, carminative, astringent, diuretic, and lotion against lice [82]. Many secondary compounds were identified in *T. nilotica*, such as phenols (nilocitin, ellagic acid, and gallic acid), flavonoids (kaempferol, tamarixetin, quercetin, isoquercitrin, flavone, and naringenin,), terpenoids, steroids, tannins, and cardiac glycosides [82,83]. On the other hand, previous phytochemical studies of the genus *Limonium* led to the isolation of flavonoids, alkaloids, tannins, carbonyl compounds, hydrocarbons, naphthoquinone, and amino acids [84,85]. Trabelsi et al. [86] reported that *L. monopetalum* was more enriched with phenolic compounds (gallic, syringic, vanillic, p-coumaric, ferulic, and transcinnamic acids) and four flavonoids (quercetin, apigenin, amentoflavone, and flavones). Therefore, this species possesses several important therapeutic properties, such as anti-dysenteric agents, antioxidant activity, antibacterial, and antifungal activities [86,87].

### 3.4. Antioxidant Activity

The selected five halophytes demonstrated a substantial decrease in DPPH• absorbance in a concentration dependent way (Table 4). The MeOH extracts of *A. macrostachyum, H. strobilaceum, T. nilotica, L. monopetalum,* and *L. pruinosum* exhibited SC_50_ values of 27.79, 28.62, 33.13, 35.72, and 37.15 mg mL^−1^, respectively. Based on the data of SC_50_ value, the ascorbic acid (standard antioxidant) showed about three-fold of the antioxidant activity than the MeOH extract of all plants (Table 4).

The halophytes growing along the seashore are subjected to a variety of abiotic stresses, including fluctuating salinity, temperature, light, nutrient, and water availability [13]. As a method of acclimation to stressful conditions, it has been shown that salinity stimulates the synthesis of phenolic compounds [88]. Additionally, when plants are exposed to salt stress, their enzymatic and non-enzymatic defense systems are induced to maintain the level of reactive oxygen species (ROS) in the plant cells [89]. The total phenolic content of *Thymus vulgaris* and *T. daenensis* was found to increase by 20% following the application of 60 mM NaCl, which boosted the antioxidant ability [88]. Our Egyptian ecospecies showed lower antioxidant activity compared to the Portuguese ecospecies of *A. macrostachyum* [76]. The higher content of phenolics of *A. macrostachyum* has been reported to be responsible for its antioxidant and reductive activities [77]. This variation in the contents of the bioactive compounds and the bioactivity could be ascribed to the effect of the environment, climate, genetics, or nutrients in the soil [31]. Additionally, seasonal variation has been reported to affect the antioxidant capacity of halophytes extract [90]. Halophytes have been reported to develop different strategies to tolerate harsh environmental conditions, such as the activation of antioxidant enzymes and proline biosynthesis [7]. In other cases, halophytes change the chlorophyll ratio and trigger the biosynthesis of antioxidant compounds, such as carotenoids, phenolics, and flavonoids [7].

In addition, halophytes can produce large quantities of other secondary metabolites, including, flavonoids, proanthocyanidins, tannins, and other antioxidant compounds [91]. These bioactive chemicals function as antioxidants, reducing the effects of oxidative stress and scavenging reactive oxygen species [51,55,57].

## 4. Conclusions

The present study revealed that studied halophytes have a substantial composition of nutritional compounds as well as minerals. The studied halophytes can be ranked according to their nutritive value as following: *H.* *strobilaceum* > *L.* *monopetalum* > *A.* *macrostachyum* > *L.* *pruinosum* > *T. nilotica*. However, the antinutritional composition, such as alkaloids and saponins must be taken into consideration. On the other side, the *A. macrostachyum* is characterized by a high content of total phenolics and total flavonoids while *H.* *strobilaceum* has been reported as a rich plant with tannins and saponin contents. Additionally, the MeOH extract of *A. macrostachyum* and *H. strobilaceum* exhibited substantial antioxidant activity comparable with ascorbic acid as a standard. The present results showed that the studied dominant halophytes could be considered as candidates for forage production or used as a green eco-friendly natural resources for bioactive compounds. A further experimental study is recommended for the evaluation of studied halophytes as non-conventional forage for different animals and evaluating their safety and sustainability.

## Figures and Tables

**Table 1:** Scientific name, life span, life from, chorotype, and habitats of the studied halophytes species.

Botanical Name	Common Name	Duration	Life Form	Chorotype	Habitat
*Arthrocnemum macrostachyum* (Moric.)K. koch.	Shenan	Perennial	Ch	ME + SA	Sd, Sf, Sm, La
*Halocnemum strobilaceum* (pall) M. Bieb.	Hatab Ahmar	Perennial	Ch	ME + IR + SA	Sf, Sm, La
*Limoniastrum monopetalum* (L.) Boiss.	Zeita	Perennial	Ch	ME	Sf, La
*Limonium pruinosum* (L.) Chaz.	Molleih	Perennial	G, He	SA	Sf, Sm, La
*Tamarix nilotica* (Ehrenb). Bunge	Tarfa	Perennial	Nph	SA + SZ	Sm, Rw, Hw, Wi, Af, Dr, La

Ch: Chamaephytes; G: Geophytes; He: Helophytes; Nph: Nanophanerophytes; ME: Mediterranean; IR: Irano-Turanian; SA: Saharo-Sindian; SZ: Sudano-Zambezian; Sd: Sand dunes; Sf: sand flats, Sm: Salt marshes; La: Lake; Rw: Railways; Hw: High ways; Wi: Waste lands; Af: Abandoned fields; Dr: Drains.

**Table 2:** Proximate composition, fiber fractionations, and mineral composition of studied halophytes.

Parameters	Halophytes Species					*LSD*_0.05_
	*A. macrostachyum*	*H. strobilaceum*	*L. monopetalum*	*L. pruinosum*	*T. nilotica*	
Moisture content	16.68 ± 0.64 ^b^	20.25 ± 0.78 ^a^	9.11 ± 0.35 ^cd^	7.13 ± 0.27 ^d^	11.24 ± 0.43 ^c^	2.74 ***
Dry matter	83.32 ± 3.20 ^b^	79.75 ± 3.07 ^b^	90.89 ± 3.50 ^a^	92.87 ± 3.57 ^a^	88.76 ± 3.41 ^a^	4.76 ***
Total ash	9.58 ± 0.64 ^a^	5.94 ± 0.23 ^b^	6.56 ± 0.25 ^b^	7.69 ± 0.30 ^b^	7.54 ± 0.29 ^b^	4.25 ***
Crude fiber (%)	17.55 ± 0.67 ^a^	22.78 ± 0.49 ^b^	7.92 ± 0.30 ^c^	8.79 ± 0.34 ^bc^	9.61 ± 0.37 ^bc^	2.74 **
Holocellulose (%)	61.36 ± 2.45 ^b^	64.68 ± 3.21 ^ab^	51.89 ± 2.11 ^c^	52.78 ± 2.18 ^c^	68.97 ± 3.52 ^a^	4.75 ***
Cellulose (%)	41.71 ± 1.65 ^b^	38.11 ± 1.89 ^c^	31.54 ± 2.07 ^e^	33.37 ± 1.08 ^d^	46.61 ± 2.84 ^a^	2.22 ***
Hemicellulose (%)	19.65 ± 0.87 ^a^	16.57 ± 0.67 ^b^	20.35 ± 0.94 ^a^	19.41 ± 0.64 ^a^	22.36 ± 0.57 ^a^	4.25 *
Lignin (%)	9.81 ± 0.07 ^d^	10.66 ± 0.12 ^c^	12.98 ± 0.5 ^b^	10.68 ± 0.23 ^c^	15.72 ± 0.53 ^a^	0.37 ***
Lipid %	1.45 ± 0.06 ^c^	5.88 ± 0.23 ^a^	1.41 ± 0.05 ^c^	1.17 ± 0.04 ^c^	2.15 ± 0.08 ^b^	0.37 ***
Crude protein %	6.88 ± 0.26 ^c^	12.36 ± 0.48 ^b^	18.00 ± 0.69 ^a^	12.81 ± 0.49 ^b^	5.97 ± 0.23 ^c^	1.61 ***
Glucose (mg g^−1^ DW)	0.63 ± 0.02 ^e^	1.66 ± 0.06 ^c^	2.65 ± 0.10 ^a^	1.36 ± 0.05 ^d^	1.88 ± 0.07 ^b^	0.09 ***
Sucrose (mg g^−1^ DW)	2.87 ± 0.11 ^b^	8.31 ± 0.32 ^a^	3.17 ± 0.12 ^b^	2.31 ± 0.09 ^b^	3.26 ± 0.13 ^b^	1.11 ***
TSS (mg g^−1^ DW)	52.55 ± 2.02 ^d^	149.51 ± 5.75 ^a^	87.30 ± 3.36 ^b^	55.05 ± 2.12 ^d^	77.10 ± 2.97 ^c^	6.65 ***
TC (mg g^−1^ DW)	323.67 ± 6.92 ^d^	354.62 ± 7.58 ^c^	371.87 ± 7.95 ^bc^	391.15 ± 8.36 ^b^	420.34 ± 8.98 ^a^	19.89 ***
NV (kcal 100 g^−1^ DW)	110.77 ± 5.32 ^b^	193.48 ± 7.32 ^a^	116.37 ± 4.69 ^c^	96.93 ± 3.58 ^d^	81.67 ± 2.69 ^e^	12.35 ***
Macro-elements (mg g^−1^ DW)						
Na^+^	27.18 ± 0.58 ^a^	20.50 ± 0.44 ^b^	18.97 ± 0.41 ^b^	20.37 ± 0.39 ^b^	23.72 ± 0.51 ^ab^	5.99 ns
K^+^	64.21 ± 1.37 ^a^	26.70 ± 0.57 ^b^	12.21 ± 0.26 ^c^	13.97 ± 0.30 ^c^	12.42 ± 0.27 ^c^	4.89 ***
Ca^2+^	47.27 ± 1.01 ^a^	32.82 ± 0.70 ^b^	14.83 ± 0.32 ^c^	15.73 ± 0.34 ^c^	15.83 ± 0.32 ^c^	9.31 ***
Mg^2+^	13.25 ± 0.28 ^a^	11.32 ± 0.24 ^ab^	8.77 ± 0.19 ^b^	9.83 ± 0.21 ^ab^	10.33 ± 0.22 ^ab^	3.63 ns

TSS: total soluble sugars, TC: Total carbohydrates, NV: nutritive value, Different superscript letters within each row showed a significant difference after Duncan’s post hoc test. * *p* < 0.05, ** *p* < 0.01, *** *p* < 0.001, ns: non-significant.

**Table 3:** Bioactive composition (mg g^−1^ DW) of the five studied halophytes.

Plant Species	Tannins	Saponins	Total Flavonoids	Alkaloids	Total Phenolics
*A. macrostachyum*	4.42 ± 0.13 ^e^	13.03 ± 0.39 ^d^	8.23 ± 0.25 ^a^	6.07 ± 0.18 ^a^	41.83 ± 1.27 ^a^
*H. strobilaceum*	22.38 ± 0.51 ^a^	22.29 ± 0.36 ^a^	7.10 ± 0.29 ^ab^	6.67 ± 0.25 ^a^	18.72 ± 0.83 ^c^
*L. monopetalum*	14.25 ± 0.39 ^c^	19.77 ± 0.20 ^c^	4.93 ± 0.08 ^bc^	7.13 ± 0.15 ^a^	17.01 ± 0.21 ^d^
*L. pruinosum*	15.81 ± 0.43 ^b^	21.10 ± 0.60 ^b^	5.26 ± 0.15 ^bc^	7.36 ± 0.22 ^a^	18.46 ± 0.52 ^c^
*T. nilotica*	11.82 ± 0.36 ^d^	6.72 ± 0.20 ^e^	4.52 ± 0.14 ^c^	3.36 ± 0.10 ^b^	22.49 ± 0.68 ^b^
*LSD_0.05_*	0.92 ***	1.12 ***	2.22 *	2.43 *	0.37 ***

Different superscript letters within each column showed a significant difference after Duncan’s post hoc test. * *p* < 0.05, *** *p* < 0.001. Data are mean values ± standard error (*n = 3*).

**Table 4:** Scavenging activity percentage of 2,2-Diphenyl-1-picrylhydrazyl (DPPH•) and the SC_50_ values by the methanolic extract of the five studied halophytes and ascorbic acid as standard.

Concentration (mg mL^−1^)	Halophytes Species				
	*A. macrostachyum*	*H. strobilaceum*	*L. monopetalum*	*L. pruinosum*	*T. nilotica*
50	71.63 ± 2.65 ^a^	58.67 ± 2.17 ^a^	66.88 ± 2.48 ^a^	65.15 ± 2.41 ^a^	70.47 ± 2.61 ^a^
40	63.81 ± 2.36 ^b^	46.58 ± 1.73 ^b^	56.62 ± 2.10 ^b^	54.89 ± 2.03 ^b^	58.21 ± 2.16 ^b^
30	52.24 ± 1.93 ^c^	39.53 ± 1.46 ^c^	43.17 ± 1.60 ^c^	41.44 ± 1.53 ^c^	46.76 ± 1.73 ^c^
20	43.63 ± 1.62 ^d^	24.33 ± 0.91 ^d^	27.97 ± 1.04 ^d^	26.24 ± 0.97 ^d^	31.56 ± 1.17 ^d^
10	32.54 ± 1.21 ^e^	17.19 ± 0.64 ^e^	20.83 ± 0.77 ^e^	19.78 ± 0.71 ^e^	24.42 ± 0.90 ^e^
5	23.94 ± 0.89 ^f^	9.40 ± 0.35 ^f^	13.04 ± 0.48 ^f^	10.81 ± 0.42 ^f^	16.63 ± 0.62 ^f^
LSD_0.05_	6.13 ***	4.29 ***	2.67 ***	4.91 ***	5.58 ***
SC_50_ (mg mL^−1^)	27.79	28.62	35.72	37.15	33.13
**Concentration** **(mg mL^−1^)**	**Ascorbic Acid**				
20	67.48 ± 1.17 ^a^				
15	58.74 ± 0.69 ^b^				
10	47.70 ± 0.47 ^c^				
5	40.71 ± 0.15 ^c^				
2.5	9.84 ± 0.07 ^d^				
1	2.85 ± 0.03 ^d^				
LSD_0.05_	8.55 ***				
SC_50_ (mg mL^−1^)	12.64				

Different superscript letters within each column showed a significant difference after Duncan’s post hoc test. *** significant at 0.001.

## Data Availability

Not applicable.

## Supplementary Materials

The following are available online, Table S1: Soil analysis of the representative habitats supporting the growth of the studied halophytes along the Deltaic Mediterranean coastal desert, Egypt.

## Sample Availability

Samples of the compounds are not available from the authors.
