# Supplementary material for: Proximate Composition, Bioactive Compounds, and Antioxidant Potential of Wild Halophytes Grown in Coastal Salt Marsh Habitats
Source: Molecules. 2021 Dec 22;27(1):28. doi: 10.3390/molecules27010028 (PMC8746247; doi:10.3390/molecules27010028)
Supplement: Supplementary file 1 [file molecules-27-00028-s001.zip › molecules-1518514-supplementary.pdf]

molecules

Article

Proximate Composition, Bioactive Compounds, and Antioxidant Potential of Wild Halophytes Grown in Coastal Salt Marsh Habitats

Yasser A. El-Amier [1,*], Walid Soufan [2], Khalid F. Almutairi [2], Nouf S. Zaghloul [3] and Ahmed M. Abd-ElGawad [1,2,*]

[1] Department of Botany, Faculty of Science, Mansoura University, Mansoura 35516, Egypt
[2] Plant Production Department, College of Food & Agriculture Sciences, King Saud University, P.O. Box 2460 Riyadh 11451, Saudi Arabia; wsoufan@ksu.edu.sa (W.S.); almutairik@ksu.edu.sa (K.F.A.)
[3] Bristol Centre for Functional Nanomaterials, HH Wills Physics Laboratory, Tyndall Avenue, Bristol, BS8 1FD, UK; nouf.zaghloul@bristol.ac.uk
* Correspondence: yasran@mans.edu.eg (Y.A.E.-A.); aibrahim2@ksu.edu.sa (A.M.A.-E.); Tel.: 00201017229120 (Y.A.E.-A.); 00966562680864 (A.M.A.-E.)

Supplementary Materials

Table S1. Soil analysis of the representative habitats supporting the growth of the studied halophytes along the Deltaic Mediterranean coastal desert, Egypt.

Soil variables	*A. macrostachyum* (n=9)	*H. strobilaceum* (n=7)	*L. monopetalum* (n=4)	*L. pruinosum* (n=4)	*T. nilotica* (n=8)	$LSD_{0.05}$
Sand (%)	90.72±0.72*	94.26±1.04	94.05±1.32	92.93±1.77	92.03±0.67	11.09ns
Silt (%)	5.88±0.36	3.47±0.59	3.90±1.00	4.85±1.28	4.98±0.38	6.60ns
Clay (%)	3.40±0.37	2.27±0.46	2.05±0.36	2.23±0.50	3.00±0.31	4.82ns
Porosity (%)	43.68±0.88	42.66±1.46	38.85±2.21	43.60±2.29	40.85±1.02	13.08ns
WHC (%)	37.90±0.63	38.34±0.77	37.13±0.85	37.08±1.24	39.81±0.61	8.33ns
pH	9.21±0.06	9.30±0.05	9.45±0.09	9.43±0.06	9.18±0.04	0.55ns
EC (mS cm^{-1})	3.22±0.32	2.43±0.38	0.75±0.95	1.90±0.32	1.35±0.13	3.67ns
$CaCO_3$ (%)	4.95±0.23	3.14±0.20	9.09±1.59	8.75±1.45	3.85±0.15	6.12ns
Organic carbon (%)	0.70±0.03	0.60±0.03	0.62±0.05	0.68±0.05	0.75±0.03	0.37ns
Cl^- (%)	0.29±0.03	0.22±0.04	0.07±0.01	0.19±0.03	0.12±0.01	0.36ns
SO_4^{2-} (%)	0.18±0.02	0.15±0.02	0.05±0.01	0.11±0.02	0.08±0.01	0.22ns
HCO_3^- (%)	0.20±0.02	0.16±0.03	0.05±0.01	0.13±0.02	0.09±0.01	0.24ns
Na^+ (mg $100g^{-1}$)	221.85±24.23	166.95±26.74	51.75±6.55	128.23±21.57	90.42±8.85	268.36ns
K^+ (mg $100g^{-1}$)	34.54±3.89	25.41±4.42	9.07±1.23	21.65±3.44	15.16±1.50	43.42ns
Ca^{2+} (mg $100g^{-1}$)	60.69±6.49	46.94±7.65	15.15±1.90	39.23±6.35	26.55±2.61	74.26ns
Mg^{2+} (mg $100g^{-1}$)	21.71±2.16	18.93±3.42	7.02±0.90	19.05±3.28	11.43±1.15	28.47ns

*Mean values ± standard error. Different letter within each row means values with significant difference after Duncan's post hoc test ($p < 0.05$). $LSD_{0.05}$: least significant difference at probability level of 0.05. ns: non-significant, WHC: water holding capacity, and EC: electrical conductivity.
